# Effectiveness of biological nurturing on early breastfeeding problems: a randomized controlled trial

**DOI:** 10.1186/s13006-020-00261-4

**Published:** 2020-04-05

**Authors:** Mariarosa Milinco, Laura Travan, Adriano Cattaneo, Alessandra Knowles, Maria Vittoria Sola, Enrica Causin, Caterina Cortivo, Maura Degrassi, Francesca Di Tommaso, Giuseppa Verardi, Laura Dipietro, Maria Piazza, Sabrina Scolz, Martina Rossetto, Luca Ronfani, for the Trieste BN (Biological Nurturing) Investigators, Graziella Andreassich, Antonietta Antonino, Silvia Bidoli, Maria Bonelli, Pierpaolo Brovedani, Jenny Bua, Donatella Bubnich, Maria Chignoli, Valentina Ciacci, Rosaria Cirillo, Aurora Colombera, Gabriele Cont, Elisabetta Crismani, Cristiana Delli Carri, Enrica Dovier, Filippa Favuzza, Claudia Ferro, Claudia Flaiban, Chiara Garofalo, Marina Giugovaz, Simona Guerrera, Erika Imbrogno, Izabella Kis, Valentina Liut, Carla Marandola, Miriana Marzullo, Antonella Mottola, Silvia Nider, Matteo Pavan, Annalisa Pelaschiar, Emma Persic, Maria Petruzzelli, Rosa Piccirillo, Angela Pirrone, Tiziana Pucci, Marzia Rabusin, Davide Scattorin, Tanja Sedmak, Patrizia Semenzato, Giulia Silvestri, Valentina Speranza, Cristina Sustersich, Lorella Tormena, Flora Torretta, Antonella Trappan, Virginia Tropiano, Chiara Zanon, Dolores Zollia

**Affiliations:** 1 Clinical Epidemiology and Public Health Research Unit, Institute for Maternal and Child Health - IRCCS “Burlo Garofolo”, Trieste, Italy; 2 Neonatal Intensive Care Unit, Institute for Maternal and Child Health - IRCCS “Burlo Garofolo”, Trieste, Italy; 3 Azienda Sanitaria Universitaria Giuliano Isontina (ASU GI), Trieste, Italy; 4 Obstetrics and Gynecology Department, Institute for Maternal and Child Health - IRCCS “Burlo Garofolo”, Trieste, Italy; 5 Life and Health Sciences Department, University of Trieste, Trieste, Italy

**Keywords:** Breastfeeding, Biological nurturing, Breast problems, Exclusive breastfeeding, Public health

## Abstract

**Background:**

Biological nurturing is a neurobehavioral approach to breastfeeding support that encourages women to breastfed in a relaxed, laidback position. This approach has the potential to reduce breast problems (e.g., sore nipples), making good latch easier and thus facilitating the initiation of exclusive breastfeeding. However, its effects have not been adequately investigated in a real-life situation. The aim of this randomized controlled trial was to assess the effectiveness of biological nurturing, compared to usual hospital practices, on the frequency of breast problems and on the prevalence of exclusive breastfeeding at discharge from the maternity ward, after 1 week, and at one and 4 months.

**Methods:**

Open randomized parallel controlled trial carried out in a third level maternity ward (IRCCS Burlo Garofolo, Trieste, Italy) between March and December 2018. Two-hundred eight women who planned to give birth at the hospital and who expressed the intention to breastfeed were enrolled during pregnancy and randomized to receive breastfeeding support following either the biological nurturing approach or the usual care protocol based on the WHO/UNICEF 20-h course, in use at the hospital. The primary study outcome was the incidence of breast problems during hospital stay, defined as the presence of one or more of the following outcomes, collected separately: sore nipples, cracked nipples, engorgement and mastitis. The primary analysis was performed by intention to treat. The follow up lasted 4 months.

**Results:**

One hundred eighty eight out of 208 women (90.3%) were included in the analysis, 90 allocated to the biological nurturing group and 98 to the usual care group. At discharge from the maternity ward, biological nurturing significantly reduced the risk of breast problems (Relative risk [RR] 0.56, 95% Confidence Interval [CI] 0.40, 0.79), including cracked (RR 0.42, 95% CI 0.24, 0.74) and sore nipples (RR 0.59, 95% CI 0.40, 0.88). No statistically significant difference was observed for exclusive breastfeeding at discharge and up to 4 months. No adverse events occurred.

**Conclusions:**

The biological nurturing approach applied in the real-life situation of a third level hospital was effective in preventing breast problems.

**Trial registration:**

Clinicaltrials.gov NCT03503500. Date of First Submission: 28 March 2018.

## Background

Breastfeeding (BF), and in particular exclusive breastfeeding (EBF), is an effective way to promote population health. Extensive and solid evidence shows that BF and EBF play a major role in ensuring optimal health, growth and development of children, providing benefits also for women, families, health services and society in general [1]. Despite this evidence and the strong recommendation to breastfeed babies promoted by almost 30 years of WHO/UNICEF Baby Friendly Hospital Initiatives (BFHI) [1–4], EBF rates still fall short of national and international goals [5–7]. The development of interventions to increase EBF rates is therefore a priority for researchers as well as policy makers. Among the main determinants that can be addressed, the quality of care and support provided by health services to women in the perinatal period seems particularly relevant [8–11]. Adequate support for the initiation of EBF is not always granted to women in hospital settings, especially when time and skills are limited [12]. The support to initiate EBF recommended by the BFHI, as described in the WHO/UNICEF 20-h course, may be demanding, requiring one or more assessments of feeds and an evaluation of latch before providing advice [13].

In recent years, a growing body of evidence on primitive neonatal reflexes has paved the way for new interventions [14–17]. The so-called biological nurturing (BN) approach, or laid-back breastfeeding, has the potential to reduce breast problems (e.g., sore nipples) making a good latch easier and thus facilitating the initiation and establishment of exclusive breastfeeding. Biological Nurturing is a neurobehavioral approach to BF that encourages the mother to breastfeed in a relaxed, laidback position, with the baby lying prone on her chest and gravity ensuring the largest possible contact between the baby’s body and the mother’s chest and abdomen. This position opens up the mother’s body and promotes the baby’s movements through the activation of 20 primitive neonatal reflexes that stimulate breastfeeding [14, 15]. Neurophysiological studies have shown that, through this approach, infants instinctively learn how to reach the nipple, latch and suckle, and mothers are able to activate the neonatal reflexes through instinctive behaviours [15, 16]. The BN approach is simple and requires no specific position or particular procedure; on the contrary, the BFHI approach relies on specific indications on “correct” positioning and latch provided by health personnel to the breastfeeding mother. Moreover, in the position with the woman sitting upright, only a limited number of primitive neonatal reflexes are elicited and neither the baby nor the mother feels comfortable [17].

The effectiveness of BN, however, has not been adequately studied in hospital settings, where several factors may limit its application (the physical setting itself, the resistance of health workers to new interventions, women’s pre-established beliefs on breastfeeding). The results of a small, unpublished trial carried out in France, have provided preliminary support to the hypothesis of the potential benefits of the BN approach. Thirty-two women with latch-on problems in the first 2 days after birth were randomized to receive support with BN vs usual care. In the BN group, there was less need for supplementation (19% vs 26%) and no women stopped breastfeeding vs nine in the usual care group [18].

Our randomized controlled trial aimed at assessing the effectiveness of BN, compared to usual hospital practices, on the frequency of breast problems and on the prevalence of EBF, at discharge, after 1 week, and at one and 4 months.

## Methods

### Study design

This was an open randomized parallel controlled trial carried out in the maternity ward of IRCCS Burlo Garofolo, a research institute and third level hospital located in Trieste, Italy. The study was coordinated by the Clinical Epidemiology and Public Health Research Unit of the Institute. The protocol was approved by the Clinical Research Office of IRCCS Burlo Garofolo and by the Regional Bioethics Committee of the Friuli Venezia Giulia Region (CEUR-2018-Sper-003-BURLO, 06/02/2018), and was registered on ClinicalTrials.gov (number NCT03503500). Written informed consent was obtained from all women enrolled in the study. The trial received no commercial funding.

### Participants

Women enrolled during pregnancy and breastfeeding infants.

Inclusion criteria:

- women who planned to give birth at IRCCS Burlo Garofolo and who expressed the intention to breastfeed, identified during the visit for their 3rd antenatal ultrasound scan (30/32 weeks of gestation).

Exclusion criteria:

- presence of maternal problems with potential negative impact on BF (e.g. severe cardiovascular problems, severe obesity as defined by body mass index above 32, hypertensive disorders);
- antenatal diagnosis of foetal complex diseases (i.e., congenital pulmonary adenomatoid malformation);
- twin pregnancy.

The need for admission to the Intensive Care Unit, at birth or during hospital stay, for either the newborn or the mother, and the appearance of neonatal jaundice requiring phototherapy, were reasons for exclusion after randomization as, in these cases, the management of feeding differs from normal practice.

### Randomization and masking

Patients were randomly assigned in fixed blocks of 10 to receive BN support or usual care. The randomization list was generated using the STATA software. The randomization procedure was centralized and managed by an independent statistician (LR). Allocation concealment was ensured by the use of consecutively numbered, sealed, opaque envelopes. At enrolment, after assessment of the inclusion/exclusion criteria, the researchers opened the envelope with the lowest number and allocated the woman to the corresponding group. The enrolling researchers and the participants were unaware of the randomization list but not blinded to the study group, while the personnel in charge of the follow-up was blinded to the study group allocation (only a patient identification code was reported on the follow-up questionnaires, with no indication of the allocation group).

### Intervention

After receiving the relevant information, eligible women were randomized to receive breastfeeding support according to one of the following:

1. the BN approach (experimental group). At randomization, the Italian version of the “Biological nurturing: laid-back breastfeeding for mothers” video (Geddes Productions, Los Angeles, CA), providing detailed information on BN, was given to women with the recommendation to watch it before delivery. During their stay in the maternity ward, women were supported by staff to breastfeed in a relaxed, laidback position, with their babies lying prone on their chests, to allow for the largest possible contact between the baby’s body and the mother’s chest and abdomen.
2. the usual care protocol, based on the WHO/UNICEF 20-h course, in use at IRCCS Burlo Garofolo (control group) [13]. At randomization, the Italian version of the “Breast is best” video (National Resource Centre for Breastfeeding, Oslo), providing detailed information on breastfeeding based on the WHO/UNICEF approach, was given to women with the recommendation to watch it before delivery. During their stay in the maternity ward, mothers were shown how to breastfeed in the sitting upright position and helped to attach their babies to the breast correctly following the WHO/UNICEF 20-h course.

Women received the videos in the form of DVDs, email link or USB pen-drive.

After delivery, to avoid or reduce spill-over between groups, women were allocated to different rooms based on the randomization group. All healthcare staff normally involved in maternity ward activities (nurses and midwifes) took care of women in both groups but were instructed to provide differentiated BF support depending on room allocation.

Regardless of allocation, all women received the standard post-partum care provided by the hospital:

- skin to skin contact in delivery room soon after birth;
- breastfeeding on demand and rooming-in 24 h a day, during maternity ward stay.

All the staff working in the maternity ward is periodically trained on the WHO/UNICEF 20-h course. In addition to this, a brief 6-h course on BN was developed by a peer counselor (MM) to train maternity ward staff for the specific purpose of this study. The course included: presentation of the study (60 min), physiology of lactation (60 min), BN and innate reflexes (90 min), BN video (60 min), role play and final discussion (90 min).

### Outcomes

The primary outcome was the incidence of breast problems during hospital stay. This variable was defined in the study protocol as the presence of one or more of the following outcomes, collected separately: sore nipples (without fissures), cracked nipples (presence of a fissure on the nipple), engorgement, and mastitis (with or without infection and/or abscess).

The secondary study outcomes were:

- incidence of breast problems at 7, 30 and 120 days after hospital discharge;
- EBF during hospital stay, in the 24 h prior to hospital discharge, and at 7, 30 and 120 days after hospital discharge; EBF was defined according to the World Health Organization definition, as infants receiving only breast milk, from their mother or from a wet nurse, through breastfeeding or breast milk expression, and no other liquids or solids, except for drops of syrups with nutritional supplements or medicines [19];
- use of nipple shields during hospital stay and at 7, 30 and 120 days after hospital discharge;
- degree of maternal satisfaction with breastfeeding during hospital stay and at 30 and 120 days after hospital discharge; this outcome was assessed using a specific subscale of the Maternal Breastfeeding Evaluation Scale (MBFES) [20], a 30-item scale that measures mothers’ satisfaction with the breastfeeding experience using a 5-point Likert scale (1 = strongly disagree to 5 = strongly agree). For the purpose of this study, only the Maternal Enjoyment/Role Attainment Subscale (questions 1, 2, 6, 9, 11, 12, 16, 17, 18, 20, 21, 23, 25, and 30) was used;
- feasibility of the BN approach in a hospital setting, assessed based on the proportion of women who refused consent to participate or withdrew from the study, on the shifting between groups, and on the proportion of women who received an intervention different from the one originally allocated.

In addition to the above outcomes, data were also collected on the main characteristics of the women (nationality, family type, education, occupation), on previous pregnancies and on feeding practices with previous children. Furthermore, at each follow-up, information was gathered on whether the mother or the child had experienced any health problems (other than breast problems) since the last contact, on attendance to postpartum courses offered by the community health services, and on return to work.

The study outcomes relating to hospital stay were recorded at discharge by nurses or neonatologists on call, who were not directly involved in the study. Follow-up data at 7, 30 and 120 days were gathered by phone. For the evaluation of breastfeeding at discharge and at 7, 30 and 120 days, the 24-h recall period recommended by WHO was used [21]. For the other outcomes, the recall period covered the entire period since the last contact.

### Statistical analysis

This study was designed as a superiority trial. We hypothesized a benefit for the BN group in terms of reduction of breast problems at discharge (primary study outcome). Available data from our Institute, indicated a current incidence of breast problems at discharge of 40% with usual BF support. We calculated that, in order to detect a reduction in the incidence of breast problems from 40 to 20% with the adoption of the BN approach, the number of subjects enrolled in each group would have to be 94 (for a total of 188 subjects), with alpha at 0.05 and beta at 0.20 (study power 80%). Considering a potential loss of 10% after randomization and the application of exclusion criteria at birth, we decided to enroll 10 additional women per group for a total of 208 women. The primary study analysis was conducted following the intention-to-treat principle. A modified intention-to-treat analysis was carried out, that included all the infants with known outcome in the group in which they were originally randomized, irrespective of the intervention they subsequently received. A per-protocol analysis, including only the infants with known outcome who received the intervention originally allocated, was also carried out. Categorical variables are presented as absolute numbers, proportions and relative risk (RR) with 95% confidence intervals (CI); continuous variables as median and interquartile range (IQR). For the primary outcome, the results for both the composite variable and the single variables are presented. The differences between the two study groups were analysed with the chi-square test or the Fisher’s exact test, as appropriate. The same type of analysis was used to evaluate the secondary outcomes. The differences in satisfaction scores between the two groups were analysed with the non-parametric Mann-Whitney U test. The use of nonparametric tests was justified by the non-normal distribution of data, evaluated both visually and with the Kolmogorov-Smirnov test. Differences with *p* value < 0.05 were considered statistically significant.

## Results

**Fig. 1:**
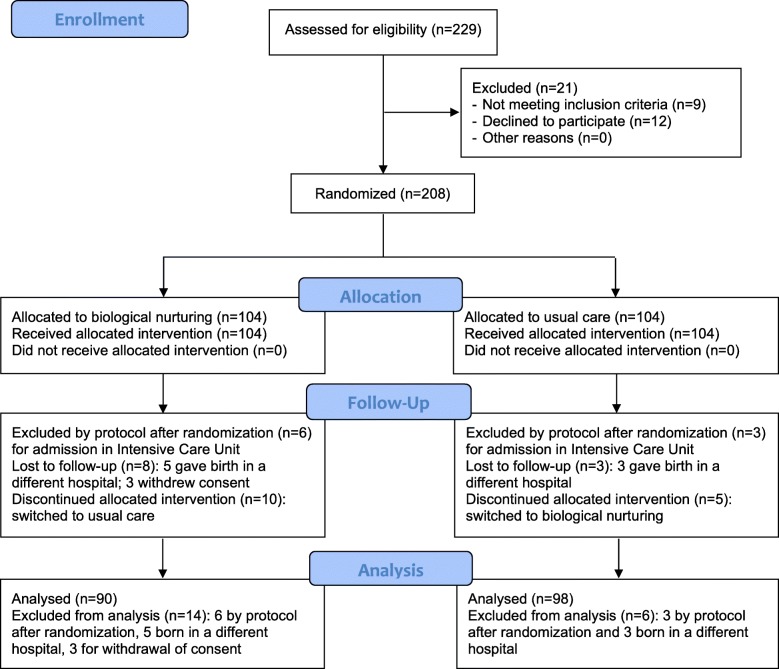
CONSORT study flow chart

The recruitment of pregnant women began on 28 March 2018. The intervention started in June 2018 with the first birth, the follow up ended in December 2018. A total of 229 women were assessed for eligibility. Twenty-one (9.2%) did not meet the eligibility criteria (eight did not fulfill the inclusion criteria, one presented exclusion criteria) or refused to participate (12 refused randomization for personal reasons) (Fig. 1).

Two hundred and eight women were randomized, 104 to BN and group 104 to usual care. Six mother-newborn pairs in the BN group and three in the usual care were excluded by protocol after randomization for admission to the Intensive Care Unit. Eight women in the BN group were lost to follow-up (five gave birth in a different hospital; three withdrew consent after delivery) and three in the usual care group (gave birth in a different hospital). Furthermore, 10 women originally allocated to the BN group received usual care, and five allocated to usual care received Biological Nurturing According to the intention-to-treat principle, the women who shifted to the other group were considered as belonging to their original randomization group (Fig. 1).

The baseline characteristics of the two groups were similar (Table 1).

**Table 1:** - General characteristics of the study sample

	Biological Nurturing (***n*** = 90)	Usual care (***n*** = 98)
Italian nationality, *n* (%)	76 (84.4%)	83 (84.7%)
Monoparental family, *n* (%)	2 (2.2%)	2 (2.0%)
High school or university degree, *n* (%)	77 (85.6%)	88 (89.8%)
Mother employed, *n* (%)	66 (73.3%)	80 (81.6%)
Previous children, *n* (%)	36 (40.0%)	40 (40.8%)
Previous children breastfed, *n* (%)*	31 (86.1%)	38 (95.0%)
Problems with previous breastfeeding, *n* (%)**	18 (58.1%)	22 (57.9%)
Antenatal course frequency, *n* (%)§	65 (73.9%)	74 (75.5%)
Vaginal birth, *n* (%)	77 (85.6%)	79 (80.6%)
Gestational age at birth, weeks, median (IQR)	40.0 (39.0–40.3)	39.0 (39.0–40.0)
Birthweight, grams, median (IQR)	3352.5 (3087.5–3702.5)	3345.0 (3058.8–3630.0)

* percentages are calculated on 76 women with previous children

** percentages are calculated on 69 women with previous breastfeeding experience

§ data available for 186/188 women

**Fig. 2:**
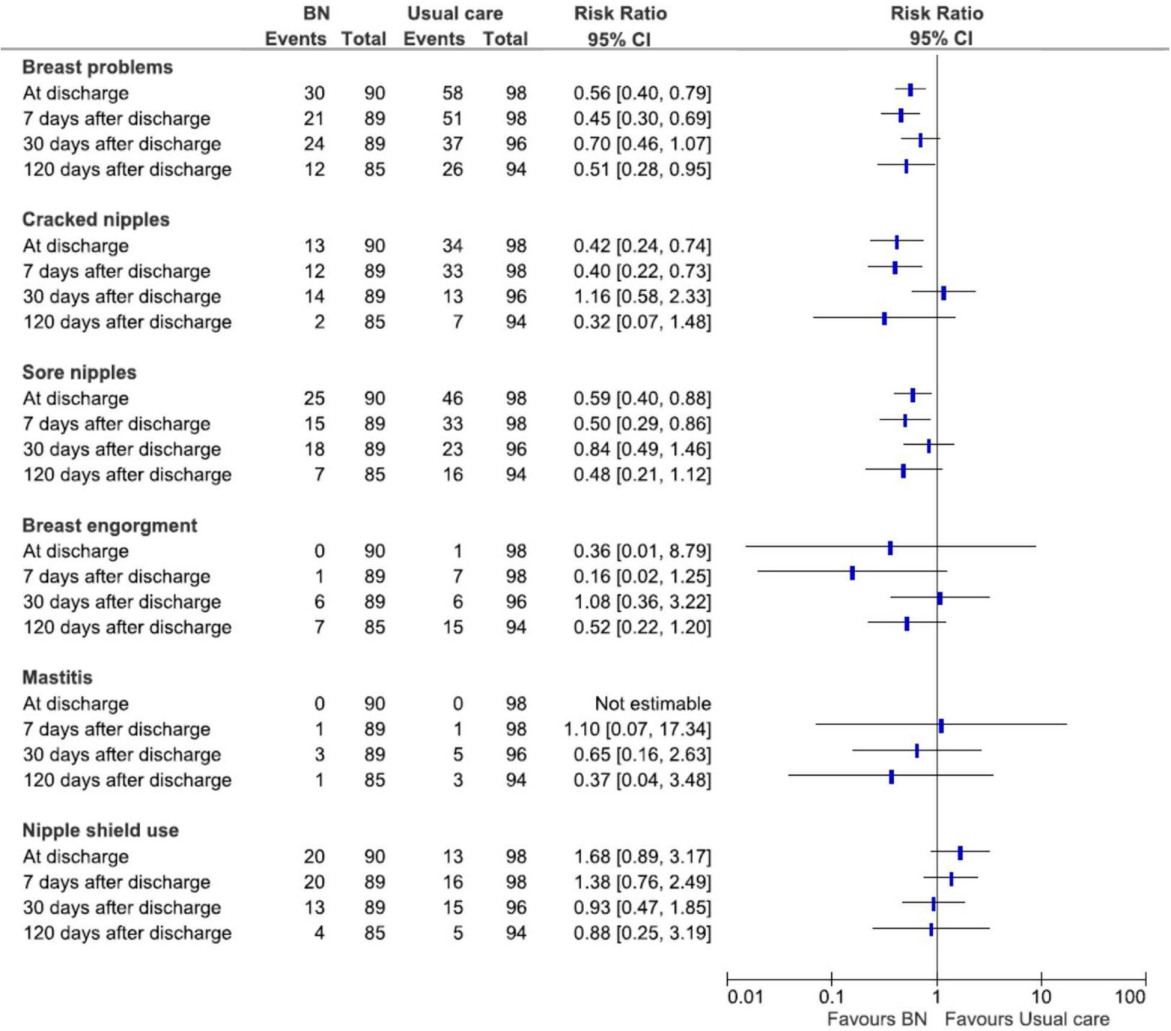
Study results, intention-to-treat analysis

The results of the intention-to-treat analysis (Fig. 2 and Tables 1–4 in Additional file 1) show that at discharge from the maternity ward, BN significantly reduced the risk of overall breast problems (RR 0.56, 95% CI 0.40, 0.79; number needed to treat 4, 95% CI 3, 9), cracked nipples (RR 0.42, 95% CI 0.24, 0.74; number needed to treat 5, 95% CI 4, 12) and sore nipples (RR 0.59, 95% CI 0.40, 0.88; number needed to treat 6, 95% CI 3, 18). No cases of mastitis, and only one of breast engorgement, were reported. No statistically significant differences between the two groups were observed for nipple shield use, and EBF during hospital stay and at discharge (24-h recall).

These results were substantially confirmed at 7 days after discharge. Notably, seven cases of breast engorgement occurred in the usual care group vs one in the BN group (RR 0.16, 95% CI 0.02, 1.25; number needed to treat 17, 95% CI 9, 213).

No statistically significant difference between groups was observed at 30 days after discharge. Compared to the data at 7 days after discharge, the number of breast problems at 30 days had decreased in the usual care group and increased slightly in the BN group. There were no differences between the two groups regarding attendance of post-partum courses, return to work, presence of maternal and child health problems, which are variables potentially associated to breastfeeding rates and breast problems.

At the end of the 120-day follow-up period, women in the BN group presented a statistically significant reduction of the risk of breast problems (RR 0.51, 95% CI 0.28, 0.95¸ number needed to treat 8, 95% CI 4, 49). No other statistically significant difference was seen between groups.

**Fig. 3:**
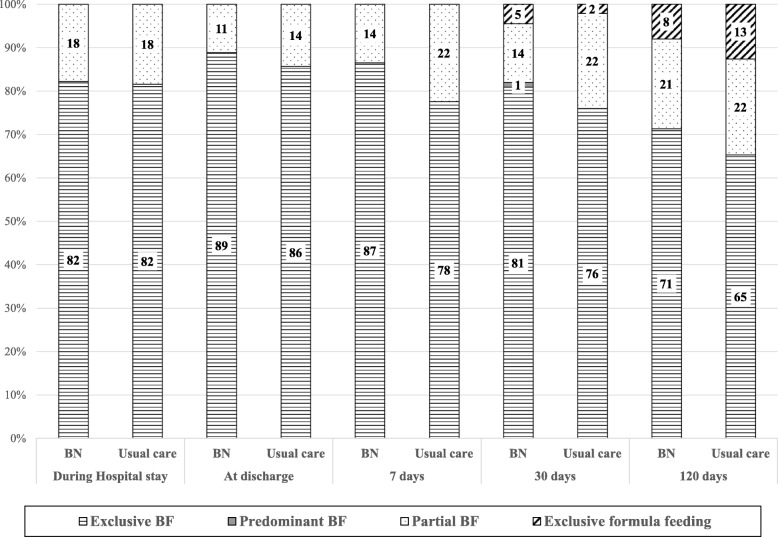
Patterns of infant feeding during the study

Figure 3 shows the trends in infant feeding during the study. No statistically significant difference between groups was seen at any of the follow-ups, despite there being a trend towards higher rates of EBF in the BN group.

The results of the per-protocol analysis fully confirmed those of the intention-to-treat analysis (Additional file 2).

Table 2 shows the results of the analysis of maternal satisfaction with breastfeeding. No difference between groups was observed.

**Table 2:** Overall maternal satisfaction concerning breastfeeding

	Biological Nurturing		Usual care		*p*
	*N*	Median (IQR)	*N*	Median (IQR)	
During hospital stay	90	64.0 (58.0 to 68.0)	98	65.0 (58.0 to 69.0)	0.42
30 days after discharge	89	61.0 (53.0 to 67.0)	96	64.0 (54.3 to 68.8)	0.12
120 days after discharge	87	62.0 (53.0 to 67.0)	95	64.0 (55.0 to 69.0)	0.25

Regarding feasibility of the BN approach, only 5% of women (12/229) refused to participate (Fig. 1); five women in BN vs three in usual care gave birth in a different hospital; three women in BN vs none in usual care withdrew consent; 10 women in BN vs five in usual care shifted to the other intervention.

No adverse event occurred.

## Discussion

To our knowledge, and with the exception of the already mentioned small, unpublished study from France [18], this is the first randomized trial on the effectiveness of the BN approach. Our study shows that, in the real-life setting of a third level hospital, and compared to the WHO/UNICEF 20-h course approach, the application of BN significantly reduces the incidence of breast problems during hospital stay and after discharge. These results are particularly impressive for cracked nipples, with a relative risk reduction of 58% at discharge (RR 0.42, 95% CI 0.24, 0.74) and of 60% (RR 0.40, 95% CI 0.22, 0.73) at 7 days after discharge. Breast problems are the main reason for earlier than desired cessation of breastfeeding [22, 23]. Papers on the management of breast problems abound in the literature, including systematic reviews, but studies on prevention are scarce and do not provide effective solutions [24–26]. Causes of breast problems are multiple and, among these, incorrect positioning and attachment are always prominent, followed by tongue tie, infection, palatal anomaly, flat or inverted nipples, and mastitis. While early diagnosis and treatment remain crucial to deal with some of these causes, our results show that BN has the potential to prevent approximately half of the breast problems during initiation and establishment of BF. These results may be explained by a higher proportion of successful latching and self-attachment with the laid-back position. Previous neurophysiological studies highlight the importance of primitive neonatal reflexes for breastfeeding and of the maternal posture for their release. When mothers sit upright, gravity pulls the baby away from the mother’s body. The release of infant innate reflexes is limited when mothers hold their babies close to counteract gravity. Conversely, when mothers lie back, innate reflexes become purposeful and help infants find the breast and latch well [14, 15].

Our study did not find statistically significant differences in EBF rates from birth to 4 months. In the assessment of this outcome due consideration must be given to the role played by the community health services of the city of Trieste, which were awarded the “Baby Friendly Community Initiative” label by UNICEF in 2014. The competent support provided by these highly skilled health professionals to mothers with BF difficulties is likely to have reduced the differences in EBF rates between the two groups. Indeed, a possible example of this is the relevant reduction in breast problems in the usual care group between seven and 30 days after discharge (Fig. 2 and Additional file 1), which is when mothers are assisted by the community health services. Despite this, EBF rates were consistently, though not significantly, higher in the BN compared to the usual care group, particularly at 7 days after discharge. The lack of statistical significance of the differences in EBF rates between groups may be real, but could be also due to the fact that the study was powered to identify differences in the primary outcome. A trial with greater power and a larger sample would be required to demonstrate that the BN approach is effective also on EBF. On the other hand, the application of the breastfeeding position recommended by WHO/UNICEF in the older version of the 20-h course approach [27], does not appear to lead to higher EBF rates and/or lower incidence of breast problems, especially in settings where EBF rates are already relatively high [28–30].

Our study showed a relatively high rate of nipple shield use. However, studies carried out in Denmark and Sweden described similar rates (about 18–20% at the beginning of breastfeeding and 10% at 3 months) [31, 32]. The use of nipple shields remains controversial as published research does not provide evidence for safety or effectiveness [33].

In our study, only a very limited number of women refused to participate, mostly for personal reasons. Three women, all in the BN group, withdrew their consent after delivery, suggesting a possible link with the allocation to BN. Furthermore, more women originally allocated to the BN group shifted to usual care after delivery than vice versa (10 vs 5), for reasons that we did not investigate. On the whole, however, the shift was very limited, women responded positively to BN and the degree of satisfaction with the BF experience did not differ from that of the usual care group. BN was safe and no adverse event occurred. Taken together, these results support the feasibility of the BN approach in hospital settings.

The major strength of our study is the fact that it was carried out with a pragmatic approach in a real-life situation. No particular restrictions were applied at enrolment through exclusion criteria. Almost all women attending their 3rd antenatal ultrasound scan and delivering in the hospital were enrolled (primiparae and multiparae, with vaginal and cesarean section delivery). During hospital stay, they were assisted and supported by the healthcare personnel routinely involved in post-partum care. This staff only received a short 6-h training on BN that included notions on primitive maternal and neonatal reflexes and their role in establishing EBF, and a practical demonstration of how the semi-reclined maternal position allows for these reflexes to be more easily released. The implementation of the BN approach did not require special equipment or supplies but only minor adjustments to ordinary maternity hospital beds. Based on this experience, we believe that the intervention we propose can easily be implemented in other maternity wards.

Among the limitations of the study are the loss to follow-up of some study subjects and the lack of total compliance with the allocated intervention, which could be explained by the fact that both study groups were admitted to the same ward, albeit in different rooms, that the staff was new to this intervention, as well as by women’s pre-established beliefs on breastfeeding. Nevertheless, the 10% increase in sample size and the adoption of an intention-to-treat approach to the primary statistical analysis, allowed us to overcome these limitations. We cannot exclude that the lack of blinding may have influenced the study results, particularly during maternity ward stay. However, the researchers in charge of the follow-ups were blinded to the study group allocation. Given that the results at 7 days fully confirm those at discharge, we hypothesize that the lack of blinding had little or no effect on the outcomes. The prevalence of EBF in the population enrolled in the study was higher than expected based on the epidemiological data available for our Institute. We cannot exclude that this finding is a consequence of the study and of the greater attention given to breastfeeding during the study period. However, the prevalence of breast problems in the usual care group was as expected, excluding the risk of a selection bias. All the healthcare personnel of the maternity ward received a short ad hoc 6-h course on BN before starting the study, but no WHO/UNICEF course reinforcement was provided. This is because, prior to this study, none of the health personnel had ever practiced BN but all of them had received, in the course of their professional career, periodic training on BF support according to the WHO/UNICEF 20-h training course.

## Conclusions

In this study, the promotion of the BN approach in the real-life setting of a third level hospital showed to be effective in preventing breast problems during initiation and establishment of breastfeeding. Should these results be confirmed in other settings, an intervention that halves the incidence of breast problems has the potential to become an important public health measure for the promotion of breastfeeding.

## Supplementary information

**Additional file 1.** Intention to treat analysis.

**Additional file 2.** Per protocol analysis.

## Data Availability

The dataset used during the current study is available from the corresponding author on reasonable request.

## Abbreviations

BFHI: Baby Friendly Hospital Initiatives
BN: Biological nurturing
CI: Confidence interval
EBF: Exclusive breastfeeding
IQR: Interquartile range
MBFES: Maternal Breastfeeding Evaluation Scale
RR: Relative risk
WHO: World Health Organization

## References

[1] Victora CG, Bahl R, Barros AJD, França GV, Horton S, Krasevec J (2016). Breastfeeding in the 21st century: epidemiology, mechanisms, and lifelong effect. Lancet.

[2] World Health Organization (2003). Global Strategy for Infant and Young Child Feeding.

[3] American Academy of Pediatrics (2012). Breastfeeding and the use of human Milk. Pediatrics.

[4] Ministero della Salute*.* Linee di indirizzo nazionali sulla protezione, la promozione ed il sostegno dell’allattamento al seno. Gazzetta Ufficiale della Repubblica Italiana, Rome, 2008.

[5] Bagci Bosi AT, Eriksen KG, Sobko T, Wijnhoven TM, Breda J (2016). Breastfeeding practices and policies in WHO European region member states. Public Health Nutr.

[6] Cai X, Wardlaw T, Brown DW (2012). Global trends in exclusive breastfeeding. Int Breastfeed J.

[7] ISTAT (2014). Anno 2013 Gravidanza, parto e allattamento.

[8] Gerd AT, Bergman S, Dahlgren J, Roswall J, Alm B (2012). Factors associated with discontinuation of breastfeeding before 1 month of age. Acta Paediatr.

[9] Erkkola M, Salmenhaara M, Kronberg-Kippilä C, Ahonen S, Arkkola T, Uusitalo L (2010). Determinants of breast-feeding in a Finnish birth cohort. Public Health Nutr.

[10] McInnes RJ, Chambers JA (2008). Supporting breastfeeding mothers: qualitative synthesis. J Adv Nurs.

[11] McFadden A, Gavine A, Renfrew MJ, Wade A, Buchanan P, Taylor JL (2017). Support for healthy breastfeeding mothers with healthy term babies. Cochrane Database Syst Rev.

[12] Hunter L, Magill-Cuerden J, McCourt C (2015). Oh no, no, no, we haven′t got time to be doing that’: challenges encountered introducing a breast-feeding support intervention on a postnatal ward. Midwifery.

[13] World Health Organization and UNICEF. Baby-friendly hospital initiative: revised, updated and expanded for integrated care. Section 3. Breastfeeding promotion and support in a baby-friendly hospital: a 20-hour course for maternity staff. WHO and UNICEF, 2009. 23926623

[14] Colson SD, Meek JH, Hawdon JM (2008). Optimal positions for the release of primitive neonatal reflexes stimulating breastfeeding. Early Hum Dev.

[15] Colson S (2012). Biological nurturing: the laid-back breastfeeding revolution. Midwifery Today Int Midwife.

[16] Klaus MH, Kennel JK (1976). Maternal-Infant Bonding: The impact of early separation of loss on family development.

[17] Colson S (2010). What happens to breastfeeding when mothers lie back? Clinical applications of biological nurturing. Clin Lact.

[18] Gadoy D*.* Breastfeeding naturally. Grenoble, France: Thesis submitted to DIULHAM; 2016.

[19] World Health Organization (2008). Indicators for assessing infant and young child feeding practices: part 1 definitions.

[20] Leff EW, Jefferis SC, Gagne MP (1994). The development of the maternal breastfeeding evaluation scale. J Hum Lact.

[21] World Health Organization (2010). Indicators for assessing infant and young child feeding practices: part 2 measurements.

[22] Li R, Fein SB, Chen J, Grummer-Strawn LM (2008). Why mothers stop breastfeeding: mothers’ self-reported reasons for stopping during the first year. Pediatrics.

[23] Odom E, Li R, Scanlon K, Perrine CG, Grummer-Strawn LM (2013). Reasons for earlier than desired cessation of breastfeeding. Pediatrics.

[24] Morland-Schultz K, Hill PD (2005). Prevention of and therapies for nipple pain: a systematic review. J Obstet Gynecol Neonatal Nurs.

[25] Niazi A, Rahimi VB, Soheili-Far S, Askari N, Rahmanian-Devin P, Sanei-Far Z (2018). A systematic review on prevention and treatment of nipple pain and fissure: are they curable?. Aust J Pharm.

[26] Dennis CL, Jackson K, Watson J (2014). Interventions for treating painful nipples among breastfeeding women. Cochrane Database Syst Rev.

[27] UNICEF/WHO (1993). Breastfeeding Management and Promotion in a Baby-Friendly Hospital - An 18-Hour Course For Maternity Staff.

[28] Henderson A, Stamp G, Pincombe J (2001). Postpartum positioning and attachment education for increasing breastfeeding: a randomized trial. Birth.

[29] Forster D, McLachlan H, Lumley J, Beanland C, Waldenström U, Amir L (2004). Two mid-pregnancy interventions to increase the initiation and duration of breastfeeding: a randomized controlled trial. Birth.

[30] de Oliveira LD, Giugliani ER, do Espirito Santo LC, França MC, Weigert EM, Kohler CV (2006). Effect of intervention to improve breastfeeding technique on the frequency of exclusive breastfeeding and lactation-related problems. J Hum Lact.

[31] Kronborg H, Foverskov E, Nilsson I, Maastrup R (2017). Why do mothers use nipple shields and how does this influence duration of exclusive breastfeeding?. Matern Child Nutr.

[32] Ekström A, Abrahamsson H, Eriksson RM, Mårtensson BL (2014). Women's use of nipple shields-their influence on breastfeeding duration after a process-oriented education for health professionals. Breastfeed Med.

[33] McKechnie AC, Eglash A (2010). Nipple shields: a review of the literature. Breastfeed Med.

