# Supplementary material for: Effectiveness of biological nurturing on early breastfeeding problems: a randomized controlled trial
Source: Int Breastfeed J. 2020 Apr 5;15:21. doi: 10.1186/s13006-020-00261-4 (PMC7132959; doi:10.1186/s13006-020-00261-4)
Supplement: Supplementary file 1 — Additional file 1. Intention to treat analysis. [file 13006_2020_261_MOESM1_ESM.docx]

**Additional file 1. Intention to treat analysis**

**Additional file 1 Table 1. Evaluation of study outcomes at discharge, intention to treat analysis**

|  | **BN**  **(n=90)** | **Usual care**  **(n=98)** | **Relative Risk**  **(95% CI)** | **p** |
| --- | --- | --- | --- | --- |
| Breast problems (primary study outcome), n (%) | 30 (33.3%) | 58 (59.2%) | 0.56 (0.40 to 0.79) | <0.001 |
| Cracked nipples, n (%) | 13 (14.4%) | 34 (34.7%) | 0.42 (0.24 to 0.74) | 0.001 |
| Sore nipples, n (%) | 25 (27.8%) | 46 (46.9%) | 0.59 (0.40 to 0.88) | 0.007 |
| Breast engorgement, n (%) | 0 | 1 (1.0%) | 0.36 (0.01 to 8.79) | 1.00^#^ |
| Mastitis, n (%) | 0 | 0 | - | - |
| Nipple shield use, n (%) | 20 (22.2%) | 13 (13.3%) | 1.68 (0.89 to 3.17) | 0.11 |
| Exclusive breastfeeding during hospital stay, n (%) | 74 (82.2%) | 80 (81.6%) | 1.01 (0.88 to 1.15) | 0.92 |
| Exclusive breastfeeding in the 24 hours prior to discharge, n (%) | 80 (88.9%) | 84 (85.7%) | 1.04 (0.93 to 1.16) | 0.52 |

^#^ Fisher’s exact test

**Additional file 1 Table 2. Evaluation of study outcomes 7 days after discharge, intention to treat analysis**

|  | **BN**  **(n=89)*** | **Usual care**  **(n=98)** | **Relative Risk**  **(95% CI)** | **p** |
| --- | --- | --- | --- | --- |
| Breast problems, n (%) | 21 (23.6%) | 51 (52.0%) | 0.45 (0.30 to 0.69) | <0.001 |
| Cracked nipples, n (%) | 12 (13.5%) | 33 (33.7%) | 0.40 (0.22 to 0.73) | 0.001 |
| Sore nipples, n (%) | 15 (16.9%) | 33 (33.7%) | 0.50 (0.29 to 0.86) | 0.009 |
| Breast engorgement, n (%) | 1 (1.1%) | 7 (7.1%) | 0.16 (0.02 to 1.25) | 0.07^#^ |
| Mastitis, n (%) | 1 (1.1%) | 1 (1.0%) | 1.10 (0.07 to 17.34) | 1.00^#^ |
| Nipple shield use, n (%) | 20 (22.5%) | 16 (16.3%) | 1.38 (0.76 to 2.49) | 0.29 |
| Exclusive breastfeeding, n (%) | 77 (86.5%) | 76 (77.6%) | 1.12 (0.98 to 1.28) | 0.11 |

* 1 woman lost to follow-up

^#^ Fisher’s exact test

**Additional file 1 Table 3. Evaluation of study outcomes at 30 days after discharge, intention to treat analysis**

|  | **BN**  **(n=89)*** | **Usual care**  **(n=96)^§^** | **Relative Risk**  **(95% CI)** | **p** |
| --- | --- | --- | --- | --- |
| Breast problems, n (%) | 24 (27.0%) | 37 (38.5%) | 0.70 (0.46 to 1.07) | 0.09 |
| Cracked nipples, n (%) | 14 (15.7%) | 13 (13.5%) | 1.16 (0.58 to 2.33) | 0.67 |
| Sore nipples, n (%) | 18 (20.2%) | 23 (24.0%) | 0.84 (0.49 to 1.46) | 0.54^#^ |
| Breast engorgement, n (%) | 6 (6.7%) | 6 (6.3%) | 1.08 (0.36 to 3.22) | 0.89 |
| Mastitis, n (%) | 3 (3.4%) | 5 (5.2%) | 0.65 (0.16 to 2.63) | 0.54 |
| Nipple shield use, n (%) | 13 (14.6%) | 15 (15.6%) | 0.93 (0.47 to 1.85) | 0.85 |
| Exclusive breastfeeding, n (%) | 72 (80.9%) | 73 (76.0%) | 1.06 (0.91 to 1.24) | 0.42 |
| Post-partum course attended, n (%) | 60 (67.4%) | 64 (66.7%) | 1.01 (0.83 to 1.24) | 0.91 |
| Back to work, n (%)** | 3 (4.6%) | 3 (3.8%) | 1.22 (0.25 to 5.82) | 1.00^#^ |
| Maternal health problems^, n (%) | 12 (13.5%) | 10 (10.4%) | 1.29 (0.59 to 2.85) | 0.52 |
| Children’s health problems, n (%) | 10 (11.2%) | 13 (13.5%) | 0.83 (0.38 to 1.80) | 0.64 |

* 1 woman lost to follow-up

**^§^** 2 women lost to follow-up

** for 144 employed women in follow-up (65 in BN group and 79 in usual care group)

^#^ Fisher’s exact test

^ other than breast problems

**Additional file 1 Table 4. Evaluation of study outcomes at 120 days after discharge, intention to treat analysis**

|  | **BN**  **(n=87)*** | **Usual care**  **(n=95)*** | **Relative Risk**  **(95% CI)** | **p** |
| --- | --- | --- | --- | --- |
| Breast problems, n (%)**^§^** | 12 (14.1%) | 26 (27.7%) | 0.51 (0.28 to 0.95) | 0.03 |
| Cracked nipples, n (%)**^§^** | 2 (2.4%) | 7 (7.4%) | 0.32 (0.07 to 1.48) | 0.12 |
| Sore nipples, n (%)**^§^** | 7 (8.2%) | 16 (17.0%) | 0.48 (0.21 to 1.12) | 0.08 |
| Breast engorgement, n (%)**^§^** | 7 (8.2%) | 15 (16.0%) | 0.52 (0.22 to 1.20) | 0.12 |
| Mastitis, n (%)**^§^** | 1 (1.2%) | 3 (3.2%) | 0.37 (0.04 to 3.48) | 0.62^#^ |
| Nipple shield use, n (%)**^§^** | 4 (4.7%) | 5 (5.3%) | 0.88 (0.25 to 3.19) | 1.00^#^ |
| Exclusive breastfeeding, n (%) | 62 (71.3%) | 62 (65.3%) | 1.09 (0.90 to 1.33) | 0.39 |
| Post-partum course attended, n (%) | 62 (71.3%) | 61 (64.2%) | 1.11 (0.91 to 1.36) | 0.31 |
| Back to work, n (%)** | 11 (16.9%) | 17 (21.5%) | 0.79 (0.40 to 1.56) | 0.49 |
| Maternal health problems^, n (%) | 5 (5.7%) | 6 (6.3%) | 0.91 (0.29 to 2.88) | 0.87 |
| Children’s health problems, n (%) | 4 (4.6%) | 3 (3.2%) | 1.46 (0.34 to 6.32) | 0.61 |

* 3 women lost to follow-up

**^§^** 2 women in BN and 1 in usual care group excluded since they were not breastfeeding at 1 month after discharge at 1 month

^#^ Fisher’s exact test

** for 144 employed women in follow-up (65 in BN group and 79 in usual care group)

^ other than breast problems
