# Supplementary material for: Effectiveness of biological nurturing on early breastfeeding problems: a randomized controlled trial
Source: Int Breastfeed J. 2020 Apr 5;15:21. doi: 10.1186/s13006-020-00261-4 (PMC7132959; doi:10.1186/s13006-020-00261-4)
Supplement: Supplementary file 2 — Additional file 2. Per protocol analysis. [file 13006_2020_261_MOESM2_ESM.doc]

**Additional file 2. Per protocol analysis**

**Additional file 2 Figure 1. Study results, per protocol analysis**


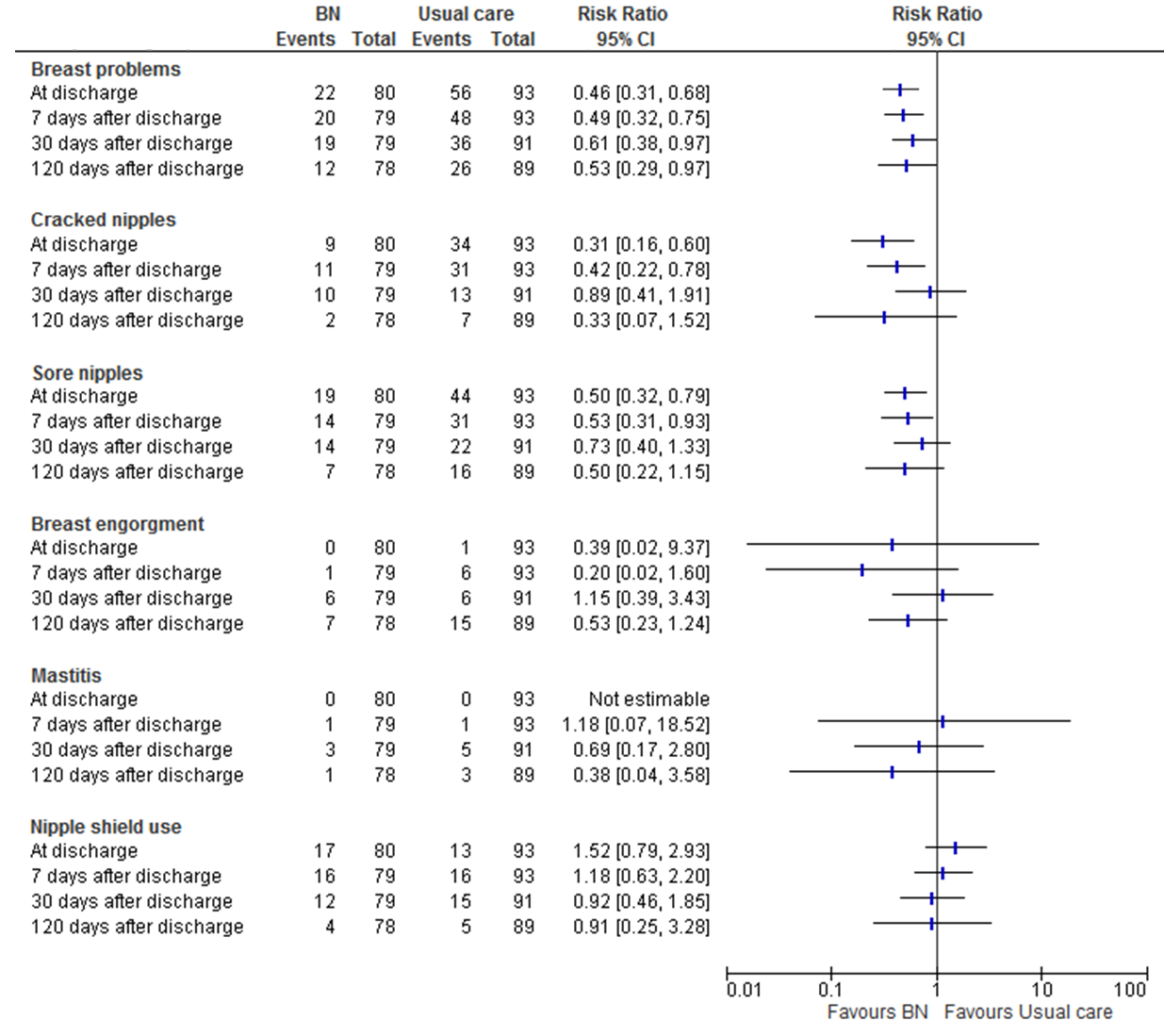


**Additional file 2 Table 1. Evaluation of study outcomes at discharge, per protocol analysis**

|  | **BN**  **(n=80)** | **Usual care**  **(n=93)** | **p** |
| --- | --- | --- | --- |
| Breast problems (primary study outcome), n (%) | 22 (27.5%) | 56 (60.2%) | <0.001 |
| Cracked nipples, n (%) | 9 (11.3%) | 34 (36.6%) | <0.001 |
| Sore nipples, n (%) | 19 (23.8%) | 44 (47.3%) | 0.001 |
| Breast engorgement, n (%) | 0 | 1 (1.1%) | 1.00^#^ |
| Mastitis, n (%) | 0 | 0 | - |
| Nipple shield use, n (%) | 17 (21.3%) | 13 (14.0%) | 0.21 |
| Exclusive breastfeeding during the hospital stay, n (%) | 67 (83.8%) | 75 (80.6%) | 0.60 |
| Exclusive breastfeeding at discharge, n (%) | 73 (91.3%) | 79 (84.9%) | 0.21 |

^#^  Fisher exact test

**Additional file 2 Table 2. Evaluation of study outcomes 7 days after discharge, per protocol analysis**

|  | **BN**  **(n=79)** | **Usual care**  **(n=93)** | **p** |
| --- | --- | --- | --- |
| Breast problems, n (%) | 20 (25.3%) | 48 (51.6%) | <0.001 |
| Cracked nipples, n (%) | 11 (13.9%) | 31 (33.3%) | 0.003 |
| Sore nipples, n (%) | 14 (17.7%) | 31 (33.3%) | 0.02 |
| Breast engorgement, n (%) | 1 (1.3%) | 6 (6.5%) | 0.13^#^ |
| Mastitis, n (%) | 1 (1.3%) | 1 (1.1%) | 1.00^#^ |
| Nipple shield use, n (%) | 16 (20.3%) | 16 (17.2%) | 0.61 |
| Exclusive breastfeeding | 70 (88.6%) | 72 (77.4%) | 0.05 |

^#^ Fisher exact test

**Additional file 2 Table 3. Evaluation of study outcomes 30 days after discharge, per protocol analysis**

|  | **BN**  **(n=79)** | **Usual care**  **(n=91)** | **p** |
| --- | --- | --- | --- |
| Breast problems, n (%) | 19 (24.1%) | 36 (39.6%) | 0.03 |
| Cracked nipples, n (%) | 10 (12.7%) | 13 (14.3%) | 0.76 |
| Sore nipples, n (%) | 14 (17.7%) | 22 (24.2%) | 0.30 |
| Breast engorgement, n (%) | 6 (7.6%) | 6 (6.6%) | 0.80 |
| Mastitis, n (%) | 3 (3.8%) | 5 (5.5%) | 0.73^#^ |
| Nipple shield use, n (%) | 12 (15.2%) | 15 (16.5%) | 0.82 |
| Exclusive breastfeeding | 65 (82.3%) | 70 (76.9%) | 0.39 |

^#^ Fisher exact test

**Additional file 2 Table 4. Evaluation of study outcomes 120 days after discharge, per protocol analysis**

|  | **BN**  **(n=79)** | **Usual care**  **(n=90)** | **p** |
| --- | --- | --- | --- |
| Breast problems, n (%)**^§^** | 12 (15.4%) | 26 (29.2%) | 0.03 |
| Cracked nipples, n (%)**^§^** | 2 (2.6%) | 7 (7.9%) | 0.18^#^ |
| Sore nipples, n (%)**^§^** | 7 (9.0%) | 16 (18.0%) | 0.09 |
| Breast engorgement, n (%)**^§^** | 7 (9.0%) | 15 (16.9%) | 0.13 |
| Mastitis, n (%)**^§^** | 1 (1.3%) | 3 (3.4%) | 0.62^#^ |
| Nipple shield use, n (%)**^§^** | 4 (5.1%) | 5 (5.6%) | 1.00^#^ |
| Exclusive breastfeeding | 60 (75.9%) | 58 (64.4%) | 0.10 |

^#^  Fisher exact test

**^§^** 1 women in BN and 1 in usual care group excluded since they did not breastfeed at 1 months
